# Time-Dependent Charge Carrier Transport with Hall Effect in Organic Semiconductors for Langevin and Non-Langevin Systems

**DOI:** 10.3390/nano12244414

**Published:** 2022-12-10

**Authors:** Seema Morab, Manickam Minakshi Sundaram, Almantas Pivrikas

**Affiliations:** College of Science, Health, Engineering and Education, Murdoch University, Perth, WA 6150, Australia

**Keywords:** organic semiconductors, Hall effect, bimolecular recombination, Langevin recombination, carrier transport

## Abstract

The time-dependent charge carrier transport and recombination processes in low-mobility organic semiconductor diodes are obtained through numerical simulations using the finite element method (FEM). The application of a Lorentz force across the diode alters the charge transport process leading to the Hall effect. In this contribution, the Hall effect parameters, such as the Hall voltage and charge carrier concentration with varying magnetic fields, are computed for both Langevin and non-Langevin type recombination processes. The results indicate the charge carrier concentration within the diode for the Langevin system is about seven and fourteen times less while the maximum amount of extracted charge is nearly five and ten times less than that in the non-Langevin system of 0.01 and 0.001, respectively. The Hall voltage values obtained for the steady-state case are similar to the non-Langevin system of $\frac{\beta }{\beta_{L}}=0.01$. However, the values obtained for the Langevin and non-Langevin systems of $\frac{\beta }{\beta_{L}}=1$ and 0.001 exhibit anomalies. The implications of these findings advance the understanding of the charge transport and Hall effect measurements in organic semiconductors that underpins the device’s performance.

## 1. Introduction

Organic semiconductors are used in a wide range of electrical and optoelectronic devices, including organic light-emitting diodes (OLEDs) [1,2,3,4], organic thin-film transistors (OTFTs) [5,6,7,8], organic photovoltaics (OPVs) [9,10,11], and organic memory [12,13]. Although inorganic semiconductors have traditionally been (and are still) the preferred materials for the modern electronic industry, research has come a long way in enhancing the performance metrics of organic-based devices since their initial appearance because of the fact that organic devices are more affordable, less complicated to manufacture, and appropriate for novel applications.

From a theoretical perspective, all of these types of devices necessitate an accurate comprehension of several factors, including charge carrier transport and its injection, the alignment of energy levels at the interface between various materials, the energy structure of organic semiconductors, the function of trap states, and charge generation and recombination. The main challenge is the fundamental processes of charge injection and conduction in organic semiconductors [14]. The organic material’s energy structure and the charge transport mechanism should both be taken into account by the conduction model. Understanding the charge-transporting ability of each material in each of the layers of the OLED is significant to create an optimum device architecture.

Time of flight (TOF), charge extraction by linearly increasing the voltage (CELIV), space-charge-limited current (SCLC), organic field-effect transistors (OFETs), and the Hall effect are popular techniques for measuring charge transport in disordered organic materials. Different trap states can be determined using the TOF approach by measuring the mobilities of holes and electrons individually. For TOF measurements, fixed negative and positive biases are employed to assess the mobilities of electrons and holes, respectively [15,16]. However, relatively thick films (>500 nm) and light illumination are needed to ensure that charge carriers cross the depletion area to reach precise mobility values.

The CELIV method is used in organic solar cells to measure the charge carrier mobility and analyse the charge extraction and recombination processes [15,17]. The CELIV method uses a triangle voltage to extract the intrinsic or photogenerated charge carriers using dark or photo-CELIV, respectively, to measure charge mobility. However, this method cannot distinguish whether the type of charge carrier is an electron or a hole but can only measure the overall charge mobility in the device.

The SCLC method is the most popular steady-state approach for measuring individual hole or electron mobilities of organic semiconductors in various device configurations. Blocking electrodes are used to generate “hole-only” and “electron-only” devices for SCLC measurements [18,19]. The actual implementation of such electrodes is not always straightforward in practice. The analysis can be inaccurate or difficult if the devices are leaky, the materials have intrinsic doping or charge traps, or both.

Charge transport dynamics in the in-plane direction can be studied using the FET method. Although the FET architecture does not require a charge-blocking layer, the crystallisation and quality of the samples must be sufficiently high to observe the desired field effect. FET measurements can identify whether the sample is n-type, p-type, or ambipolar semiconductors, in addition to measuring charge mobility in the in-plane direction. Unfortunately, the FET mobility depends on the sample’s interfacial morphology, grain size, and operating temperature. Therefore, the FET approach is more appropriate for high-crystallinity samples and purposefully altered electrode surfaces to obtain intrinsic charge mobility. Moreover, the mobilities from OFET experiments are not comparable to those from diode structures because the current is constrained to a thin layer at the interface with the dielectric layer, resulting in mobilities that are at least an order of magnitude greater than mobilities obtained by ToF [19].

The Hall effect method is a reasonable approach to evaluate the charge carrier concentration and the intrinsic defect concentration and determine whether the measured semiconductor is n-type or p-type [20]. The three methods mentioned above (CELIV, TOF, and FET) measure the mobility of charges only in a fixed direction, whereas the Hall effect technique can determine the bulk mobilities of both electron and hole carriers. Previous works have been shown for one-dimensional semiconductor devices using the above-mentioned techniques [21], whereas in this work, the Hall effect method is employed to study the charge carrier dynamics in a two-dimensional organic semiconductor diode. An in-depth analysis and better optimisation of charge transport in semiconductors is significant for improving the device performance. The present work will bring new insights on charge transport in organic semiconductors.

The paper is outlined as follows: in Section 2, a two-dimensional semiconductor model is developed using a drift–diffusion–recombination solver with a finite element method (FEM). This section also provides the parameter settings relevant to the simulations. Section 3 provides the results, highlighting all significant performance metrics (such as carrier concentration, total charge, and Hall voltages) and making a comparison with the Langevin and non-Langevin systems. Section 4 concludes with final observations.

## 2. Methods

### 2.1. Numerical Methods and Device Modelling

The organic semiconductor device model was developed using a one-dimensional drift–diffusion–recombination solver [22,23,24,25,26,27]. The device model was then extended to two dimensions to obtain the Hall effect parameters and charge carrier concentration under the influence of the magnetic field. To simplify the model, no traps, no dopants, and no thermally generated charge carriers were included in this model. Moreover, for time *t* = 0 and throughout the entire transient, the applied voltage on the electrodes was kept constant, simulating low circuit RC condition, and the electric field was homogeneous throughout the device. The continuity equations were applied to the charge carrier concentration [22,23,24,28,29]. These equations were combined with the Poisson equation to include the space charge effects. To incorporate the Hall effect in the model, two-dimensional geometry was employed, with continuity and Poisson equations applied to charge carriers in both x and y directions. According to the Hall effect, if the motion of charge carriers due to the applied electric field is in the x-direction and the applied magnetic field is in the z-direction, then the magnetic force acting on moving charges will be in the y-direction. Therefore, the magnetic force in the y-direction is represented by the equation $F_{\mathrm{magnetic}\;\mathrm{force}}=e\;\left(v_{\mathrm{dx}}\;\times \;B_{z}\right)$, where e is electron charge, $v_{\mathrm{dx}}$ is the drift velocity of electrons in the x-direction, and $B_{z}$ is the applied magnetic field in the z-direction [30,31]. All quantities were normalised and converted to dimensionless units. This was achieved by selecting reference scales for physical units such as length, voltage, and time. A method similar to Juška et al. [32,33] was used for nondimensionalisation. The dimensionless quantities were denoted by a prime symbol. For reference voltage $U_{\mathrm{ref}}=1$, the normalised voltage was $U^{\prime }=\frac{U_{\mathrm{applied}}}{U_{\mathrm{ref}}}$, such that the normalised electric field was $E^{\prime }=\frac{E\;d}{U_{\mathrm{ref}}}$. The normalised length and time scales were given by $x^{\prime }=\frac{x}{d},y^{\prime }=\frac{y}{d}\;\;\mathrm{and}\;\;t^{\prime }=\frac{t}{t_{\mathrm{tr}}}$, where d was the thickness of the semiconductor and $t_{\mathrm{tr}}$ was the transit time of charge carriers. The normalised mobility for faster carriers was $\mu_{\left(\mathrm{faster}\right)}^{\prime }=1$. The normalised charge scale represented the charge on the electrodes given by $Q^{\prime }=\frac{Q}{C\;U_{\mathrm{ref}}}$. The number density of charge carriers were normalised to charge scale $n^{\prime }=\frac{\mathrm{enSd}}{CU_{\mathrm{ref}}}$ and $p^{\prime }=\frac{\mathrm{epSd}}{CU_{\mathrm{ref}}}$, where S is the surface area of the semiconductor device. The normalisation of circuit parameters such as resistance, current, and current density were given by $R^{\prime }=\frac{\mathrm{RC}}{t_{\mathrm{tr}}},\;\;i^{\prime }=\frac{t_{\mathrm{tr}}}{CU_{\mathrm{ref}}}i,\;\mathrm{and}\;\;j^{\prime }=\frac{t_{\mathrm{tr}}}{CU_{\mathrm{ref}}}\mathrm{Sj}=\frac{t_{\mathrm{tr}}}{CU_{\mathrm{ref}}}i$, where the normalised current density and current were equal. Further, the temperature normalisation was made by $T^{\prime }=\frac{k_{B}T}{eU_{\mathrm{ref}}}$. The normalisation of the magnetic force was given by $F^{\prime }=\frac{F_{\mathrm{magnetic}}}{F_{\mathrm{electric}}}=\frac{F_{\mathrm{magnetic}}}{e\;(\frac{U_{\mathrm{ref}}}{d})}$. The dimensionless magnetic flux density was $B^{\prime }=\frac{d^{2}}{U_{\mathrm{ref}}\;t_{\mathrm{tr}}}B$ and the recombination rate was normalised to the Langevin rate by $\beta^{\prime }=\frac{\beta }{\beta_{L}}$.

The normalised model equations for the semiconductor device were:

(1) $$j_{n}^{\prime }\left(x^{\prime },t^{\prime }\right)=\mu {}_{n}^{\prime }E^{\prime }\left(x^{\prime },t^{\prime }\right)n^{\prime }\left(x^{\prime },t^{\prime }\right)+\mu {}_{n}^{\prime }T^{\prime }\frac{\partial n^{\prime }}{\partial x^{\prime }}$$

(2) $$j_{p}^{\prime }\left(x^{\prime },t^{\prime }\right)=\mu {}_{p}^{\prime }E^{\prime }\left(x^{\prime },t^{\prime }\right)p^{\prime }\left(x^{\prime },t^{\prime }\right)-\mu {}_{p}^{\prime }T^{\prime }\frac{\partial p^{\prime }}{\partial x^{\prime }}$$

(3) $$j_{n}^{\prime }\left(y^{\prime },t^{\prime }\right)=\mu {}_{n}^{\prime }F^{\prime }\left(y^{\prime },t^{\prime }\right)n^{\prime }\left(y^{\prime },t^{\prime }\right)+\mu {}_{n}^{\prime }T^{\prime }\frac{\partial n^{\prime }}{\partial y^{\prime }}$$

(4) $$j_{p}^{\prime }\left(y^{\prime },t^{\prime }\right)=\mu {}_{p}^{\prime }F^{\prime }\left(y^{\prime },t^{\prime }\right)p^{\prime }\left(y^{\prime },t^{\prime }\right)-\mu {}_{p}^{\prime }T^{\prime }\frac{\partial p^{\prime }}{\partial y^{\prime }}$$

(5) $$F_{n}^{\prime }\left(y^{\prime },t^{\prime }\right)=\mu {}_{n}^{\prime }\;\;E^{\prime }\left(x^{\prime },t^{\prime }\right)\;\;B^{\prime }\left(-z^{\prime },t^{\prime }\right)$$

(6) $$F_{p}^{\prime }\left(y^{\prime },t^{\prime }\right)=\mu {}_{p}^{\prime }\;\;E^{\prime }\left(x^{\prime },t^{\prime }\right)\;\;B^{\prime }\left(-z^{\prime },t^{\prime }\right)$$

(7) $$\frac{\partial n^{\prime }}{\partial t^{\prime }}-\frac{\partial j_{n}^{\prime }}{\partial x^{\prime }}=-r^{\prime }\left(x^{\prime },t^{\prime }\right)$$

(8) $$\frac{\partial p^{\prime }}{\partial t^{\prime }}+\frac{\partial j_{p}^{\prime }}{\partial x^{\prime }}=-r^{\prime }\left(x^{\prime },t^{\prime }\right)$$

(9) $$\frac{\partial n^{\prime }}{\partial t^{\prime }}-\frac{\partial j_{n}^{\prime }}{\partial y^{\prime }}=-r^{\prime }\left(y^{\prime },t^{\prime }\right)$$

(10) $$\frac{\partial p^{\prime }}{\partial t^{\prime }}+\frac{\partial j_{p}^{\prime }}{\partial y^{\prime }}=-r^{\prime }\left(y^{\prime },t^{\prime }\right)$$

(11) $$r^{\prime }\left(x^{\prime },t^{\prime }\right)=\beta^{\prime }\;\left(\mu_{n}^{\prime }+\mu_{p}^{\prime }\right)\;n^{\prime }\;p^{\prime }$$

(12) $$r^{\prime }\left(y^{\prime },t^{\prime }\right)=\beta^{\prime }\;\left(\mu_{n}^{\prime }+\mu_{p}^{\prime }\right)\;n^{\prime }\;p^{\prime }$$

(13) $$\frac{\partial^{2}V^{\prime }}{\partial {\left(x^{\prime }\right)}^{2}}=n^{\prime }-p^{\prime }$$

(14) $$\frac{\partial^{2}V^{\prime }}{\partial {\left(y^{\prime }\right)}^{2}}=n^{\prime }-p^{\prime }$$

(15) $$E^{\prime }=-\frac{\partial V^{\prime }}{\partial x^{\prime }}$$

(16) $$U^{\prime }\left(t^{\prime }\right)=\int_{0}^{1}E^{\prime }\left(x^{\prime },t^{\prime }\right)\;{\mathrm{dx}}^{\prime }$$

(17) $$V_{H}^{\prime }\left(t^{\prime }\right)=\int_{0}^{1}E^{\prime }\left(y^{\prime },t^{\prime }\right)\;{\mathrm{dy}}^{\prime }$$

The Poisson’s equation had boundary conditions given by:

(18) $$U^{\prime }\;\left(t^{\prime },0\right)=V^{\prime }$$

(19) $$U^{\prime }\;\left(t^{\prime },1\right)=0$$

where $V^{\prime }$ is the voltage across semiconductor and $V_{H}^{\prime }$ is the Hall voltage.

The boundary conditions for carrier transport were represented by the fluxes defined by the drift current $j_{n}^{\prime }\left(x^{\prime },t^{\prime }\right)=\mu {}_{n}^{\prime }E^{\prime }\left(x^{\prime },t^{\prime }\right)n^{\prime }\left(x^{\prime },t^{\prime }\right)$ (and similarly for holes) of Equations (1) and (2), on each injecting electrode. The voltage was applied under forward bias conditions using Equations (18) and (19), and charge carriers were injected through injecting electrodes using the Dirichlet boundary conditions. The initial conditions for electrons ($n^{\prime }$) and holes’ ($p^{\prime }$) concentrations and their time derivatives given by $\frac{\partial n^{\prime }}{\partial t^{\prime }}$ and $\frac{\partial p^{\prime }}{\partial t^{\prime }}$, respectively, were zero. The initial value for the voltage was $V^{\prime }=1$.

### 2.2. Simulation Settings

Simulations were performed using the two-dimensional geometry for the device, and the initial number of electrons and holes at each injecting electrode was 100, defined by the Dirichlet boundary conditions. The recombination rate for the Langevin materials was $\beta^{\prime }$ = 1, and for non-Langevin materials, it was $\beta^{\prime }<1$ [34,35]. The charge carriers had a balanced mobility ratio given by $\mu_{\mathrm{faster}}/\mu_{\mathrm{slower}}=1$. The temperature was selected for 1 × 10${}^{-2}$, and the magnitude of the applied magnetic field in the (−z) direction was varied from 0 to 1 [31].

The set of equations developed in the model was implemented in COMSOL Multiphysics for computation, and simulations were performed using the finite element method. For the discretization, the Lagrange shape function with the spatial frame, quadratic element order was used. The “general form PDE” in time was employed to solve the continuity and the Poisson equations. This partial differential equation (PDE) solver provided the best performance out of all standard COMSOL PDE solvers. For stability and convergence in results, the free triangular mesh was employed, with a number of mesh elements equal to 100, and the mesh size was kept “extremely fine”.

## 3. Results and Discussion

The simulation was performed with an injection of two types of charge carriers, such as electrons and holes, at the opposite electrodes of the two-dimensional semiconductor device. The voltage was applied across the semiconductor device, and as a result, the current flowed through the device with one type of charge carrier (electrons) drifting in the opposite direction. This voltage referred to the longitudinal voltage. An external magnetic field to the device generated an additional voltage called the Hall voltage ($V_{H}^{\prime }$). This voltage was a transverse voltage acting orthonormal to the direction of the flow of current. Subsequently, two processes simultaneously occurred within the semiconductor device. The first was the accumulation of charge carriers orthogonal (top and bottom edges) to the current flow within the device due to the Hall effect. This accumulation of charges increased with an increase in time. The second was the recombination of charge carriers, where charge carriers recombined either through the Langevin or non-Langevin recombination process. The numerical simulations were run to predict the total amount of extracted charge, the concentration of charge carriers, and the Hall voltage as a function of time $t^{\prime }$, position $x^{\prime }$ (or $y^{\prime }$), and magnetic field $B_{z}^{\prime }$ for different recombination coefficients $\frac{\beta }{\beta_{L}}$. The results are discussed in the three sections (Section 3.1, Section 3.2 and Section 3.3) listed below.

### 3.1. Effect of Lorentz Force on Charge Carrier Concentration

**Figure 1:**
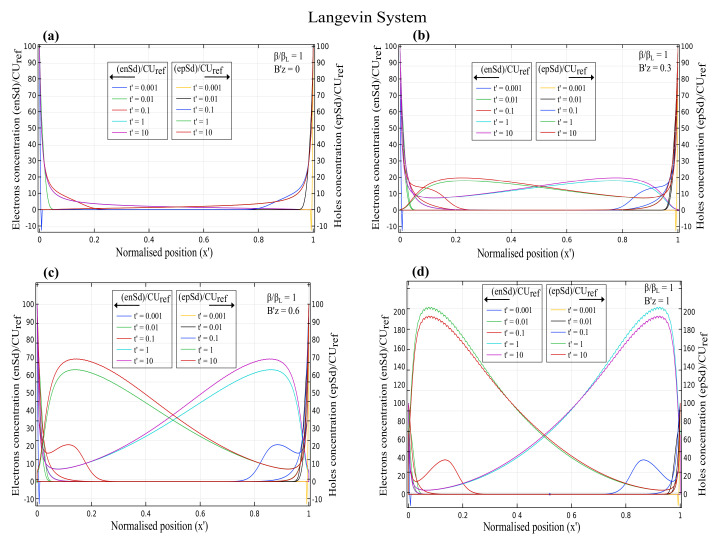
Time-dependent charge carrier concentration for electrons and holes as a function of normalised position $x^{\prime }$ with varying magnetic fields of (**a**) $B_{z}^{\prime }=0$ (**b**) $B_{z}^{\prime }=0.3$ (**c**) $B_{z}^{\prime }=0.6$ (**d**) $B_{z}^{\prime }=1$ for Langevin system with $\frac{\beta }{\beta_{L}}=1$.

**Figure 2:**
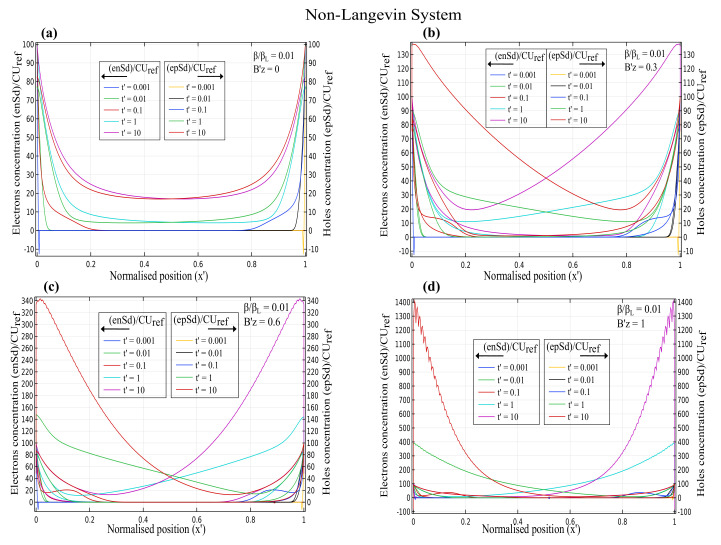
Time-dependent charge carrier concentration for electrons and holes as a function of normalised position $x^{\prime }$ with varying magnetic fields of (**a**) $B_{z}^{\prime }=0$ (**b**) $B_{z}^{\prime }=0.3$ (**c**) $B_{z}^{\prime }=0.6$ (**d**) $B_{z}^{\prime }=1$ for non-Langevin system with $\frac{\beta }{\beta_{L}}=0.01$.

**Figure 3:**
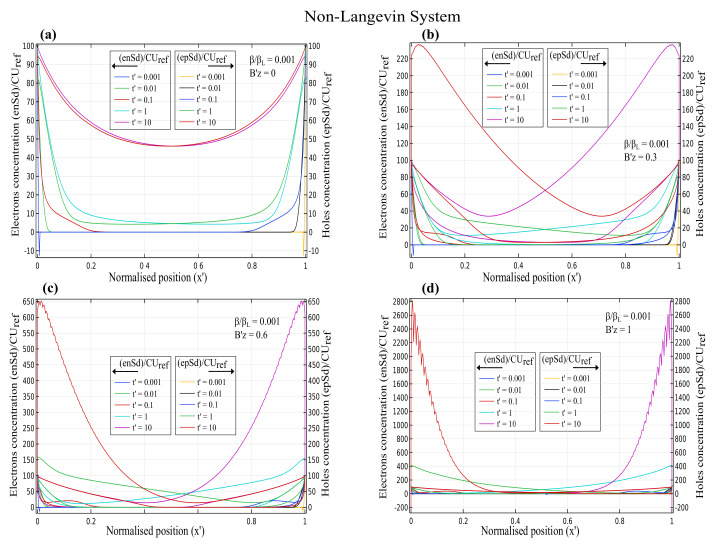
Time-dependent charge carrier concentration for electrons and holes as a function of normalised position $x^{\prime }$ with varying magnetic fields of (**a**) $B_{z}^{\prime }=0$ (**b**) $B_{z}^{\prime }=0.3$ (**c**) $B_{z}^{\prime }=0.6$ (**d**) $B_{z}^{\prime }=1$ for non-Langevin system with $\frac{\beta }{\beta_{L}}=0.001$.

The normalised charge carrier concentration for electrons ($n^{\prime }$) and holes ($p^{\prime }$) as a function of normalised position $x^{\prime }$ for various magnetic fields at different time scales is depicted in Figure 1, Figure 2 and Figure 3. The electron concentration is on the left y-axis, and the hole concentration is on the right y-axis. The magnetic field varied from $B_{z}^{\prime }$ = 0, 0.3, 0.6 and 1, and the effect of the Lorentz force on the charge concentration was analysed in both the Langevin and non-Langevin systems for times $t^{\prime }$ = 0.001, 0.01, 0.1, 1, and 10.

Figure 1a shows that for the Langevin recombination with magnetic field $B_{z}^{\prime }=0$, the electron and hole concentration decreased with an increase in the time from $t^{\prime }=0.001$ to 10. However, with increasing magnetic fields of $B_{z}^{\prime }$ = 0.3, 0.6, and 1, the concentrations decreased with an increase in the time of $t^{\prime }=0.001$ and 0.01, and then showed an irregularity at time $t^{\prime }=0.1$, with further increased the concentration for times $t^{\prime }=1$ and 10 as illustrated in Figure 1b–d. This increase in the concentration was nearly 20, 70, and 200 for $B_{z}^{\prime }$ = 0.3, 0.6, and 1, respectively.

Figure 2 demonstrates that for the non-Langevin system of $\frac{\beta }{\beta_{L}}=0.01$, the electron and hole concentrations decreased initially with increasing times $t^{\prime }=0.001$, 0.01, and 0.1, then increased for times $t^{\prime }$ = 1 and 10, and reached maximum values of about 100, 130, 340, and 1400 for magnetic fields of 0, 0.3, 0.6, and 1, respectively. Hence, the charge concentration increased with increasing magnetic fields and times.

Similar to Figure 2, Figure 3 indicates that for the non-Langevin system of $\frac{\beta }{\beta_{L}}=0.001$ with increasing times $t^{\prime }=0.001$, 0.01, and 0.1, the electron and hole concentrations initially decreased, then increased for times $t^{\prime }$ = 1 and 10, and reached maximum values of almost 100, 220, 650, and 2800 for magnetic fields of 0, 0.3, 0.6, and 1, respectively. Therefore, the charge concentration with a lesser recombination rate of 0.001 increased with increasing magnetic fields and times.

Figure 1, Figure 2 and Figure 3 illustrate that the Langevin system had a lesser concentration due to the higher recombination rate for increasing magnetic fields than in the non-Langevin system. Consequently, results showed that the maximum concentration in the Langevin system was about seven and fourteen times less than that in the non-Langevin systems of $\frac{\beta }{\beta_{L}}=0.01$ and 0.001, respectively.

### 3.2. Impact of Magnetic Field on the Total Charge

**Figure 4:**
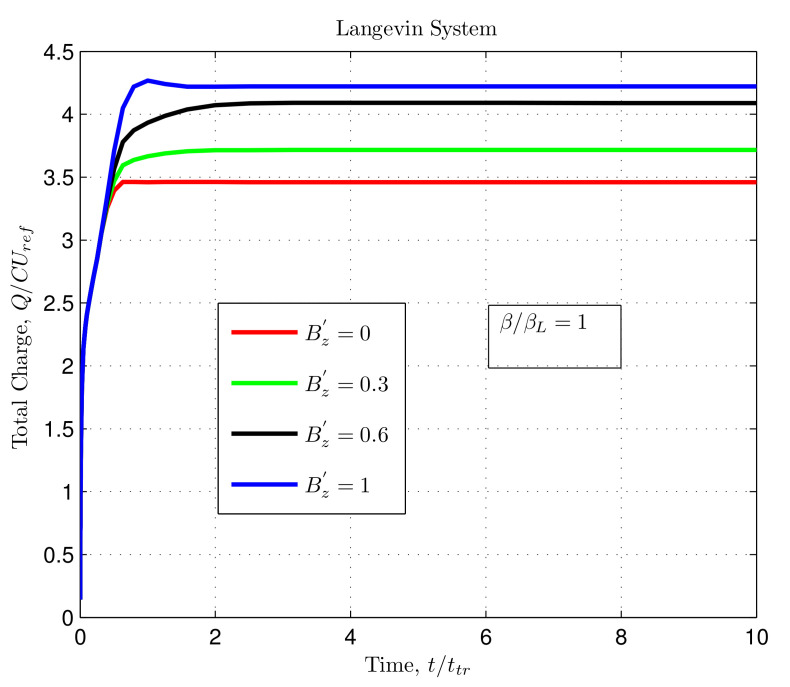
Total charge as a function of normalised time with varying magnetic fields for Langevin system with $\frac{\beta }{\beta_{L}}=1$.

**Figure 5:**
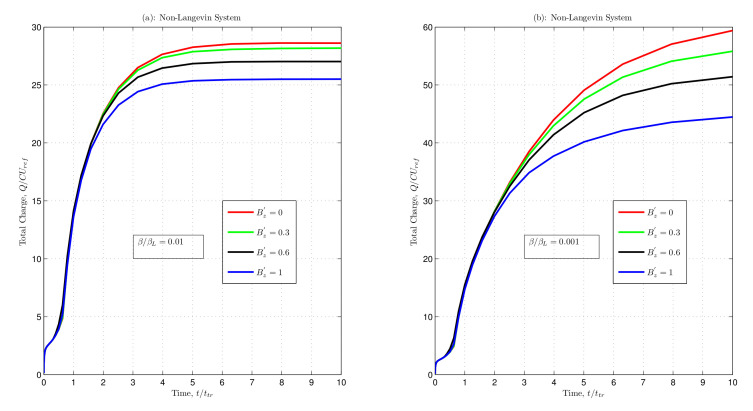
Total charge as a function of normalised time with varying magnetic fields for non-Langevin system with (**a**) $\frac{\beta }{\beta_{L}}=0.01$ and (**b**) $\frac{\beta }{\beta_{L}}=0.001$.

The magnetic field was varied to observe the dynamic behaviour of charges in both Langevin and non-Langevin systems. The impact of the magnetic field on the total charge within the semiconductor devices was examined through simulations as illustrated in Figure 4 and Figure 5.

Figure 4 shows that for a magnetic field $B_{z}^{\prime }=0$, the total charge saturated at the value of 3.46, whereas with increasing magnetic fields of $B_{z}^{\prime }$ = 0.3, 0.6, and 1, the total charge saturated to higher values of 3.71, 4.08, and 4.22, respectively. Here, the recombination coefficient $\frac{\beta }{\beta_{L}}=1$ represented the Langevin-type recombination between the charge carriers. Therefore, increasing the magnetic field increased the saturation value of the total charge.

Figure 5 demonstrates the non-Langevin recombination between the charge carriers with the recombination coefficients $\frac{\beta }{\beta_{L}}=0.01$ and 0.001. In Figure 5a, as the magnetic field increased from $B_{z}^{\prime }=0$ to 0.3, 0.6, and 1, the total charge saturated at values 28.61, 28.18, 27.02, and 25.50, respectively. Similarly, Figure 5b shows the saturation of the total charge at values 59.39, 55.81, 51.42, and 44.48, with increasing magnetic fields of $B_{z}^{\prime }=0,0.3,0.6$, and 1, respectively. Hence, increasing the magnetic field decreased the saturation value of the total charge.

Figure 4 and Figure 5 depict that the total charge increased initially with an increase in time and then readily saturated. This saturation depended almost entirely upon the applied magnetic field and recombination coefficient. However, in the non-Langevin system, less recombination occurred between the charge carriers, and more charge was extracted at lower magnetic fields (Figure 5). In contrast, a reverse condition existed for the Langevin system, where more charge extraction was possible at higher magnetic fields, even at a higher recombination rate (Figure 4). Furthermore, the results indicated that the maximum amount of extracted charge from the device in the Langevin system was about five and ten times less than that in the non-Langevin systems of $\frac{\beta }{\beta_{L}}=0.01$ and 0.001, respectively, as illustrated in the above figures.

### 3.3. Computation of Hall Voltage Using Numeric Methods

**Figure 6:**
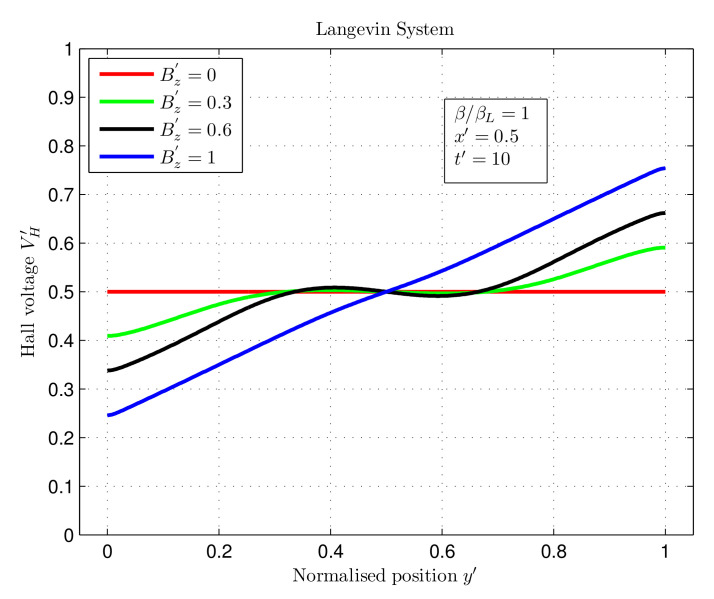
Hall voltage as a function of normalised position for varying magnetic fields in Langevin system with $\frac{\beta }{\beta_{L}}=1$.

**Figure 7:**
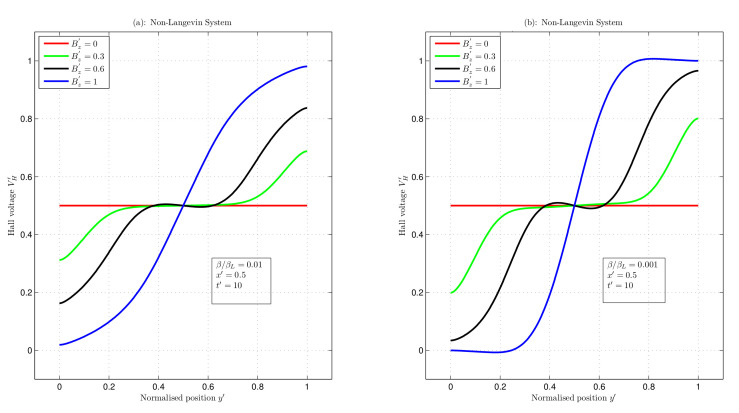
Hall voltage as a function of normalised position for varying magnetic fields in non-Langevin system with (**a**) $\frac{\beta }{\beta_{L}}=0.01$ and (**b**) $\frac{\beta }{\beta_{L}}=0.001$.

Numeric FEMs were applied to compute the Hall voltage as a function of the normalised position ($y^{\prime }$) with a fixed value of $x^{\prime }=0.5$ for different magnetic fields at time $t^{\prime }=10$, as shown in Figure 6 and Figure 7. The results were analysed for the recombination coefficients $\frac{\beta }{\beta_{L}}=1,0.01$, and 0.001 that represent Langevin and non-Langevin recombinations, respectively, between the charge carriers.

Figure 6 illustrates that for the Langevin system with a magnetic field $B_{z}^{\prime }$ = 0, the maximum and minimum voltage values were 0.5. Similarly, for magnetic fields of $B_{z}^{\prime }$ = 0.3, 0.6, and 1, the maximum and minimum voltage values were 0.59 and 0.40, 0.66 and 0.33, and 0.75 and 0.24, respectively. Therefore, the observed potential difference was 0, 0.19, 0.33, and 0.51 for increasing magnetic fields of $B_{z}^{\prime }$ = 0, 0.3, 0.6, and 1, respectively. Since the normalised equations were employed in the simulations (as discussed in the methods section), the Hall voltage values obtained were also dimensionless. Consequently, the Hall voltage increased with an increasing magnetic field.

Figure 7 depicts the non-Langevin systems with $\frac{\beta }{\beta_{L}}=0.01$ and 0.001. In Figure 7a, with increasing magnetic fields of $B_{z}^{\prime }$ = 0, 0.3, 0.6 and 1, the maximum voltage values were 0.5, 0.68, 0.83, and 0.98, respectively, and the minimum voltage values were 0.5, 0.31, 0.16, and 0.01, respectively. These values gave potential differences of 0, 0.37, 0.67, and 0.97. Moreover, in Figure 7b, the maximum and minimum voltage values were 0.5 and 0.5, 0.80 and 0.19, 0.96 and 0.03, and 1.00 and $-2.61\;\times \;10^{-4}$, giving potential differences of 0, 0.61, 0.93, and 1 for magnetic fields of $B_{z}^{\prime }$ = 0, 0.3, 0.6, and 1, respectively. Hence, these results show that increasing the magnetic field increased the Hall voltage.

Figure 6 and Figure 7 represent an increase in the Hall voltage with increasing magnetic fields. However, Table 1 shows that in the Langevin system, the Hall voltages for varying magnetic fields were less (nearly half) than those in the steady-state condition, whereas in the non-Langevin system of $\frac{\beta }{\beta_{L}}=0.01$, the Hall voltages were almost equal to those of the steady-state case. For $\frac{\beta }{\beta_{L}}=0.001$, these values were greater than those in the steady-state system. The steady-state Hall voltage equation is $V_{H}=\mu \;E_{x}\;B_{z}\;w$ [31]. This equation is normalised to obtain dimensionless units $V_{H}^{\prime }=\mu^{\prime }\;E_{x}^{\prime }\;B_{z}^{\prime }\;w^{\prime }$ with $w^{\prime }=\frac{w}{d}$, where *w* is the width of the semiconductor. The parameters $\mu^{\prime },E_{x}^{\prime }$, and $w^{\prime }$ have values equal to unity, such that the Hall voltages shown in Table 1 for the steady state are the values of the magnetic field.

## 4. Conclusions

Variations in the magnetic field and time can induce significant changes in the charge concentration and total charge, resulting in changes in the Hall voltage. In addition, Langevin and non-Langevin recombinations between charge carriers substantially affect the concentration and Hall voltage. The results demonstrated that the charge concentration in the Langevin system was about seven and fourteen times less than that in the non-Langevin systems of $\frac{\beta }{\beta_{L}}=0.01$ and 0.001. Similarly, the maximum amount of extracted charge from the device in the Langevin system was about five and ten times less than that in the non-Langevin systems of $\frac{\beta }{\beta_{L}}=0.01$ and 0.001. Moreover, in the non-Langevin system, more charge was extracted at lower magnetic fields. However, a reverse condition existed for the Langevin system, where more charge extraction was possible at higher magnetic fields, even at a higher recombination rate. Furthermore, the Hall voltages were almost equal to the steady-state case for a non-Langevin system of 0.01. However, these voltages showed anomalies for the Langevin and non-Langevin systems of 1 and 0.001. In conclusion, the significance of these results paves the way for an understanding of charge transport and Hall effect measurements in low-mobility organic semiconductors to enhance device performance.

## Figures and Tables

**Table 1:** Hall voltages in Langevin, non-Langevin, and steady-state systems.

Magnetic Field	Langevin	Non-Langevin	Non-Langevin	Steady State
$B_{z}^{\prime }$	$\frac{\beta }{\beta_{L}}=1$	$\frac{\beta }{\beta_{L}}=0.01$	$\frac{\beta }{\beta_{L}}=0.001$	$V_{H}^{\prime }$
0	0	0	0	0
0.3	0.19	0.37	0.61	0.3
0.6	0.33	0.67	0.93	0.6
1	0.51	0.97	1.0002	1

## Data Availability

Not applicable.
